# Components of high-resolution manometry that change surgical decisions

**DOI:** 10.1007/s00464-023-10161-3

**Published:** 2023-06-26

**Authors:** Georg Wiese, Carlos Delgado, Irteza Inayat, Steve Eubanks

**Affiliations:** 1 General Surgery Department, AdventHealth, Orlando, FL USA; 2 General Surgery Residency Program, AdventHealth, Orlando, FL USA; 3 Gastroenterology, AdventHealth, Orlando, FL USA; 4 2415 North Orange Avenue, Suite 400, Orlando, FL 32804 USA; 5 Department of Surgery, Loma Linda University, CA Loma Linda, USA

**Keywords:** High-resolution manometry, Fundoplication, Anti-reflux surgery, Foregut surgery

## Abstract

**Background:**

High-resolution manometry (HRM) is vital in evaluating patients for surgery at the gastroesophageal (GE) junction. Previously, we reported manometry alters surgery choices at the GE junction over 50% of the time, and its components, i.e., abnormal motility and distal contractile integral (DCI), are vital in decision-making. This single-institution retrospective study examines how HRM characteristics, reported with the Chicago classification, can alter the intended surgical plans for foregut surgery.

**Methods:**

We collected data on pre-operative symptoms for patients undergoing HRM studies from 2012 to 2016, i.e., Upper GI X-rays, 48-h pH studies, DeMeester scores, upper endoscopy, and biopsy reports. HRM results were further categorized via Chicago classification (i.e., normal or abnormal motility). The DCI was determined; Patients not seen by a surgeon were excluded. Then a single surgeon, blinded to patient identity and HRM results, determined the planned procedure. The reviewer was then exposed to the HRM results; procedural plans were revised if needed. HRM results were then evaluated to determine which factors most influenced the surgical decisions.

**Results:**

298 HRM studies were initially identified; 114 met search criteria. Overall, HRM altered the planned procedure in 50.9% of cases (*n* = 58), with abnormal motility in 54.4% (62/114) cases. Abnormal motility findings corresponded to 70.6% (41/58) of the patients in which HRM changed the surgery decision. A DCI of < 1000 was identified in only 31.6% (36/114) of all patients, but 39.7% (23/58) of cases where the surgical decision was altered. A DCI of > 5000 was identified in only 10.5% (12/114) of all patients but 10.3% (6/58) of cases with altered surgical decisions. A DCI < 1000 and abnormal motility were generally associated with a partial fundoplication.

**Conclusions:**

This study demonstrates the impact of identifying abnormal motility via the Chicago classification and factors like DCI on surgical choice at the GE junction.

Manometry is one of the most useful adjuncts to surgery decisions at the gastroesophageal (GE) junction. This tool helps reveal how the esophagus functions, thus adding physiologic knowledge to diagnose and select potential treatment methods [1–4]. Interpreting manometry has evolved to include high-resolution manometry (HRM), which allows multiple physiologic parameters to be represented in a simplified histogram that can instantly illustrate the function at the lower esophageal sphincter and the strength of contractility throughout the esophagus. The development of the Chicago classification system has further provided a systematic means to interpret HRM and allowed GE junction diseases to be classified into distinct entities, thus helping surgeons tailor their surgical approach [5].

In previous studies, we examined how manometry influenced surgical decisions. We found that the manometry results changed the planned operation at least 50.9% of the time [6]. Therefore, this current study aimed to identify which factors seen on a typical HRM exam influenced the final decision at the GE junction.

HRM with topographical temporal spatial plotting is a powerful tool for evaluating esophageal motility and reflux. Although conventional manometry was once the gold standard, HRM is distinctly advantageous because it has more sensors and offers real-time monitoring of contractile activity over the entire esophageal length [2]. When combined with impedance, manometry measures the effectiveness of bolus clearance with each swallow and can provide more easily interpreted functional insight into the swallowing mechanism of the esophagus. Additional parameters which are captured and recorded in HRM such as the distal contractile integral (DCI), integrated relaxation pressure or distal latency can also offer insight into overall esophageal pathology.

Surgeons have traditionally used manometry to evaluate patients prior to anti-reflux surgery [7–9]. When performing anti-reflux surgery it is essential in identifying achalasia. Additionally, the functional data presented by HRM can be useful for tailoring the surgical approach. For example, when one encounters failed or weak peristalsis and peristaltic defects, that patient might have less dysphagia post-surgery with partial fundoplication rather than complete. This single-institution retrospective study was designed to evaluate the impact of HRM on surgeries at the GE junction and to offer insight into what components of manometry help determine that choice.

## Materials and methods

After obtaining approval from the Institutional Review Board overseeing this study, data were gathered on all HRM studies performed at a single institution from 2012 through 2016. These studies were completed by the same gastroenterology group within a single institution and utilized the Sierra Scientific Instruments HRM devices. Manometry reports included computer-generated raw data and high-resolution color graphs displaying manometry and impedance results.

We identified 298 manometry studies performed within our institution during the selected time period. A chart review was then completed to collect data on pre-operative symptoms, manometry results, diagnosis, pre-operative assessment via Upper GI X-rays, 48-h pH studies, DeMeester scores, and upper endoscopy. The data were compiled into a single database and verified by a final reviewer.

Table 1 presents the variables collected and patient demographics. The findings of HRM were classified according to the Chicago Classification of Esophageal Motility Disorders v3.0 and v4 [5]. DCI was also recorded. To help clarify results, the patients in this study were assigned to abnormal versus normal manometry findings based on the Chicago classification. The category of Abnormal motility was assigned if > 30% failed swallows, spastic esophagus, achalasia, and elevated DCI were identified. Manometry methodology has been previously described by Kahrilas et al. [10] and Gywali [11].

**Table 1:** Demographics, pre-operative symptoms, and extent of further diagnostic workup outside of manometry

Patient demographics and symptoms	No	Percent
*(114 patients seen by a single surgeon)*		
Average age	59.7	–
Number male	39	34.2
Number female	75	65.8
*Pre-operative symptoms*		
Heartburn	79	69.3
Swallowing difficulty	51	44.7
Chest pain	36	31.6
Cough	22	19.3
Hoarseness	15	13.2
Gas/bloating	21	18.4
*Pre-operative testing*		
Number with EGD	103	90.4
Presence of short or long barrett’s esophagus	18	15.8
Presence of dysplasia	4	3.5
Number with Bravo study	63	55.3
Number with upper GI	66	57.9

Patients evaluated by the surgeon who performs the vast majority of foregut cases at a single institution were included in the final analysis. These cases ranged from first time hiatal hernia and reflux patients, to redo paraesophageal hernias and patients presenting with achalasia. Patients who underwent manometry at outside institutions prior to presentation and those receiving further evaluation and surgery at other hospitals or by a different provider were excluded. This equaled 114 of the 298 initially identified patient cases. Only the most recent perioperative dataset was included for patients with more than one manometry study.

A single surgeon analyzed the completed dataset. This surgeon was given access to the gathered data but was blinded to the patients’ identities and the manometry results. A surgical choice was then made and recorded. After the initial surgical determination, manometry results were exposed, and the procedural plans were revised as needed with subsequent correlation with the actual procedure proposed and performed for each patient. Any alterations in the surgical plans due to manometry results were then recorded. In most cases, the ultimate surgical decision is recorded as partial versus complete fundoplication.

## Results

A total of 298 HRM studies were initially identified; 114 met search criteria and were reviewed by a single surgeon. As reported previously, manometry was found to have altered the planned procedure in 50.9% of cases (58 patients). In 4.4% (5 cases), the patients met the inclusion criteria, but the clinical data available upon chart review were insufficient to determine a surgery plan regardless of manometry results.

Abnormal motility was identified by Chicago classification in 54.4% (62/114) of cases. The findings of abnormal motility corresponded to 70.6% (41/58) of the patients in which HRM changed the surgery decision. A DCI of < 1000 was identified in only 31.6% (36/114) of all patients, but 39.7% (23/58) of cases where the surgical decision was altered. A DCI of > 5000 was identified in only 10.5% (12/114) of all patients, but 10.3% (6/58) of cases where the surgical decision was altered.

Furthermore, we found that with normal motility, complete fundoplication was more likely to be chosen than partial fundoplication (35:12; 67.3%). Conversely, abnormal motility was associated with partial fundoplication in comparison to complete fundoplication (42:12; 67.7%). Similarly, DCI < 1000 was associated with more likely partial fundoplication (21:10; 58.3%). DCI > 2000 did not seem to affect a decision for partial versus complete fundoplication (14:16; 42.4%), nor did DCI > 5000 (5:5; 50%). These findings are summarized in Table 2.

**Table 2:** Breakdown of HRM and DCI

Patient manometry results (114 patients seen by a single surgeon)	No. (%) of 114 patients	HRM changed surgical plan no. (%)	Percent of the 58 where HRM changed decisions (%)	Complete fundoplication No	Partial fundoplication No	No surgery offered or other^a^ No
High-resolution manometry	114(100)	58 (50.9)		47	54	13
Abnormal motility	62 (54.4)	41 (36.0)	70.6	12	42	8
Normal motility	52 (45.6)	17 (14.9)	29.3	35	12	5
Distal contractile integral						
DCI < 1000	36 (31.6)	23 (20.2)	39.7	10	21	5
DCI 1000–1500	20 (17.5)	9 (7.9)	15.5	12	6	1
DCI < 1500 Summary	56 (49.1)	32 (28.1)	55.2	22	27	6
DCI 1500–2000	10 (8.8)	4 (3.5)	6.9	5	3	2
DCI 2000–5000	21 (18.4)	9 (7.9)	15.5	11	9	1
DCI > 5000	12 (10.5)	6 (5.2)	10.3	5	5	2
DCI = NA	16 (14)	7 (6.1)	12.1	4	10	2

Ranges of Distal Contractile integral were reported

< refers to "less than"

> refers to "greater than"

DCI = NA refers to values that were unable to be obtained during manometry

^a^Other category includes patients who were found to be candidates for fundoplication or underwent esophagectomy, roux en y, or no surgery at all

## Discussion

This study demonstrates an objective view of HRM as an integral part of the pre-operative workup for surgery at the GE junction. Furthermore, it helps delineate several components of HRM that provide valuable data for driving those surgical decisions. For instance, our data showed a strong correlation between decisions for partial fundoplication when abnormal motility was identified or if the DCI was less than 1000. Furthermore, DCI greater than 5000 did not significantly alter surgical choices in this study. These objective points helped change decisions for surgery when previous data from clinical symptoms, upper GI, or endoscopy would have led to a different surgical choice.

Our study has some limitations. For example, in utilizing manometry results from only our institution, we limited the patient population to approximately a quarter of the patients who underwent foregut surgery by our designated surgeon over the study period. This approach allowed us to utilize a more standardized and consistent set of manometry results; however, the total number of patients included in the study was less than what would otherwise be expected. Importantly, we feel that even with a limited number, these patients represent the overall population undergoing foregut surgery.

We also acknowledge that our results may be biased by using the expert opinion of only a single surgeon to analyze the results. As a counter argument, utilizing the experience of a single surgeon also gives our study consistency across all the patients evaluated. We sought to address this concern proactively by blinding the surgeon to manometry results and patient identity at the initial evaluation. Still, these results are significant, with HRM influencing more than half of surgical decisions. Further studies could verify these results by incorporating outside datasets or including additional surgeons in the analysis to evaluate for concordance. We intend to expand on this study and invite other experts to participate in correlating these results.

Additionally, one can see a high number of abnormal manometry findings in this population that was studied. Part of this may be attributed to our broad classification of abnormal motility as any manometry finding which is outside the range of normal on the Chicago classification. However, this may also be attributed to the fact that the surgeon involved is dealing not only with reflux surgery, but also a high percentage of their practice is redo fundoplications, paraesophageal hernias, and patients being worked up for achalasia.

Manometry is an essential component of surgery at the GE junction, but how it can be interpreted to help make those decisions is often up for debate. Although the debate will likely continue, this study offers insight into the decision-making process of an experienced surgeon, showing that HRM, and in particular identification of abnormal motility and defining the DCI is of great value for surgical decisions. In future studies, a closer look at other components of manometry such as integrated relaxation pressure or distal latency of the esophagus may be of additional benefit.

## Conclusion

This retrospective, single-institution study demonstrates the value of HRM and its effect on surgical decision-making at the GE junction. Furthermore, it identifies that additional factors such as low DCI and abnormal motility can also significantly influence a surgical choice.
